# Predictors of outcome in neck pain patients undergoing chiropractic care: comparison of acute and chronic patients

**DOI:** 10.1186/2045-709X-20-27

**Published:** 2012-08-24

**Authors:** Cynthia Peterson, Jennifer Bolton, B Kim Humphreys

**Affiliations:** 1 University of Zürich and Orthopaedic University Hospital Balgrist, Forchstrasse 340, 8008 Zürich, Switzerland; 2 Anglo-European College of Chiropractic, 13-15 Parkwood Road, Bournemouth, England, BH5 2DF, UK

**Keywords:** Neck pain, Chiropractic, Outcomes, Prognostic factors

## Abstract

**Background:**

Neck pain is a common complaint in patients presenting for chiropractic treatment. The few studies on predictors for improvement in patients while undergoing treatment identify duration of symptoms, neck stiffness and number of previous episodes as the strong predictor variables. The purpose of this study is to continue the research for predictors of a positive outcome in neck pain patients undergoing chiropractic treatment.

**Methods:**

Acute (< 4 weeks) (n = 274) and chronic (> 3 months) (n = 255) neck pain patients with no chiropractic or manual therapy in the prior 3 months were included. Patients completed the numerical pain rating scale (NRS) and Bournemouth questionnaire (BQ) at baseline prior to treatment. At 1 week, 1 month and 3 months after start of treatment the NRS and BQ were completed along with the Patient Global Impression of Change (PGIC) scale. Demographic information was provided by the clinician. Improvement at each of the follow up points was categorized using the PGIC. Multivariate regression analyses were done to determine significant independent predictors of improvement.

**Results:**

Baseline mean neck pain and total disability scores were significantly (p < 0.001and p < 0.008 respectively) higher in acute patients. Both groups reported significant improvement at all data collection time points, but was significantly larger for acute patients. The PGIC score at 1 week (OR = 3.35, 95% CI = 1.13-9.92) and the baseline to 1 month BQ total change score (OR = 1.07, 95% CI = 1.03-1.11) were identified as independent predictors of improvement at 3 months for acute patients. Chronic patients who reported improvement on the PGIC at 1 month were more likely to be improved at 3 months (OR = 6.04, 95% CI = 2.76-13.69). The presence of cervical radiculopathy or dizziness was not predictive of a negative outcome in these patients.

**Conclusions:**

The most consistent predictor of clinically relevant improvement at both 1 and 3 months after the start of chiropractic treatment for both acute and chronic patients is if they report improvement early in the course of treatment. The co-existence of either radiculopathy or dizziness however do not imply poorer prognosis in these patients.

## Background

Patients suffering from neck pain are second only to low back pain patients in terms of the frequency of presentation for chiropractic treatment [1-4]. For many of these patients the precise diagnosis is difficult to ascertain and thus becomes labeled ‘non-specific’ neck pain or neck pain from mechanical dysfunction [1,3-5]. Research evidence has yet to determine with clarity whether spinal manipulative therapy (SMT) or mobilization of the neck is the superior treatment for these patients [1-9] although it appears that both of these treatments have better outcomes when combined with exercise [5,10].

Manipulative therapy to the cervical spine has traditionally been considered somewhat controversial by certain health care practitioners. Recent high quality research evidence supports the relative safety of chiropractic SMT to the cervical spine with no increased risk of vertebral artery injury compared to patients seeking care from other primary medical physicians who do not manipulate the neck [11-13]. A few studies have begun to investigate specific predictors for a positive response to chiropractic SMT in neck pain patients and have identified the duration of symptoms, stiffness of the neck and the number of previous episodes of neck pain as some of the strongest predictors of an immediate positive response [7,8,14]. Therefore, the purpose of this study is to continue the research for predictors of positive outcomes in neck pain patients undergoing chiropractic treatment and to determine if these differ between acute and chronic patients.

## Methods

This is a prospective cohort study with three month follow-up. Ethics approval was obtained from the Canton of Zürich Switzerland ethics committee (EK-19/2009) and written informed consent was obtained from all patients.

### Patients

Consecutive new patients over the age of 18 with neck pain of any duration who had not undergone chiropractic or manual therapy in the prior 3 months were recruited from multiple chiropractic practices in Switzerland. Patients with specific pathologies of the cervical spine that are contraindications to chiropractic manipulative therapy, such as tumours, infections, inflammatory arthropathies, acute fractures, Paget’s disease, anti-coagulation therapy, cervical spondylotic myelopathy, known unstable congenital anomalies and severe osteoporosis, were excluded. Although the data was collected for subacute patients (symptoms between 4 and 12 weeks), for this study these patients were not used due to the small sample size (19% of total patients).

All active members of the Association of Swiss Chiropractors (ASC) (260 in total) were asked to contribute patients to this study. Notification and instructions about this study as well as the study protocol were sent to all chiropractors by email as well as discussed verbally during the annual mandatory post-graduate continuing education (CE) convention held immediately prior to the start of data collection. Workshops were also conducted during the CE convention by one of the authors on the use of outcome measures in clinical practice. It was emphasized via email and verbally that there should be no changes in the treatment methods used by the contributing chiropractors as the purpose of this study was to evaluate outcomes as would be found in routine chiropractic practice. Therefore, standardization of treatment method or treatment number was not desired. It is known however, from the data collected for the ‘Swiss Job Analysis’ study done in 2009 that the ‘diversified’ technique is applied to between 76 and 100% of chiropractic patients in Switzerland. The other commonly used treatments used include advice on the activities of daily living, trigger point therapy, therapeutic exercises and mobilization techniques [15].

### Outcome measures

The numerical rating scale (NRS) for neck pain and a separate NRS for arm pain as well as the Bournemouth Questionnaire for neck (BQN) disability, which has been translated and validated in German [16], were administered to the patient immediately prior to the first treatment by the office staff of each practice. The BQN is a multidimensional instrument covering 7 domains with each domain evaluated using an 11-point numerical rating scale (0 through 10). The seven domains include: (i) pain; (ii) disability (activities of daily living (ADL)); (iii) disability (social activities); (iv) anxiety; (v) depression; (vi) work, both inside and outside the home, fear avoidance; and (vii) locus of control. In addition to each domain, the total score (maximum 70 points) is also calculated. The BQN was shown to be much more sensitive to change compared to the German versions of the Neck Pain and Disability (NPAD) questionnaire and the Neck Disability Index (NDI) for all subscales [16].

One week after the first treatment, data from the NRS (neck), NRS (arm), Patient Global Impression of Change (PGIC) scale [17], and the BQN were collected from the patient via a short telephone interview. Similarly, these same data were collected at 1 month and 3 months after the start of treatment via telephone interviews. These telephone interviews were conducted by research assistants at the university hospital who were unknown to the patients.

### Demographic and clinical baseline data

Additional information provided by the treating chiropractor at the initial consultation included: patient age, gender, marital status, paid employment, whether or not the onset of pain was due to trauma, whether or not the patient smokes, whether or not the patient was currently taking pain medication, duration of current complaint, number of previous episodes, whether or not the patient also had signs and symptoms of cervical radiculopathy, whether or not the patient also complained of dizziness and the patient’s general health status. This information was completed on a baseline information form.

### Prognostic (predictor) variables

All variables were identified in advance and were taken from information completed by the chiropractor on the baseline information form at the first consultation and from changes in variables between baseline and the data collection time points (1 week, 1 month and 3 months). Continuous variables consisted of patient age, NRS (neck), NRS (arm), BQN scores and change scores. All other variables, with the exception of ‘number of previous episodes’ and ‘general health’, were dichotomized for ease of interpretation (yes/no or present/absent as appropriate). The ‘number of previous episodes’ was divided into 3 categories: none, 1 to 3 episodes, and 4 or more episodes. ‘General health’ was categorized as ‘good’, ‘average’, or ‘poor’ as determined by the treating chiropractor.

Patients whose symptoms were less than 4 weeks in duration were classified as ‘acute’ and those whose symptoms were longer than 12 weeks were classified as ‘chronic’. Sub-acute patients were not included in this study to make a clear distinction between acute and chronic patients. Acute vs. chronic patients were analyzed separately as previous research has shown that length of complaint is a strong predictor of outcome [7]. Moreover, there is a clear rationale to expect that predictors may well be different between acute and chronic patients given the differences in the course of their condition. This is borne out by recent research investigating prediction of outcomes in acute and chronic pain patients as separate groups [18].

### Outcomes

Outcomes were evaluated using the NRS (neck), NRS (arm) and the BQN total and sub-scale raw change (i.e. baseline minus 1 week, 1 month or 3 month) scores. For the prediction analysis the outcome ‘improvement’ was defined as scores of 1 (much better) or 2 (better) on the PGIC scale. Scores of 3 (slightly better), 4 (no change), 5 (slightly worse), 6 (worse) and 7 (much worse) were categorized as ‘not improved’.

### Statistical analysis

Baseline factors were compared between acute (symptoms < 4 weeks) and chronic (symptoms > 12 weeks) patients using the chi^2^ test for categorical variables and the unpaired t-test for continuous variables. Within patient differences for continuous variables were analysed using the paired t-test.

Multivariate regression analyses were carried out to determine statistically significant independent predictors of improvement. ‘Improvement’ as the dependent variable in the regression analysis was categorized as scores of 1 (much better) or 2 (better) on the PGIC. First, all potential predictors were separately entered into a univariate model and those significantly associated with improvement (p < 0.1) were entered in a forward stepwise multivariate model, which entered only those variables independently associated with ‘improvement’ (p <0.05). Variables in the model were checked for redundancy by noting the correlation coefficients (>0.8) between variables in the presence of the other variables in the model. The properties of the final predictive model were ascertained by calculating the sensitivity, specificity, % correctly classified, and the area under the Receiver Operator Characteristic (ROC) curve. A value of at least 70% for the area under the ROC was considered acceptable for discriminative accuracy [19]. The adjusted %R^2^ was used as the index of the percentage of the variance in the outcome explained by the model. SPSS version 17 was used for all data analyses.

## Results

### Baseline characteristics

Of the 260 active members of the Association of Swiss Chiropractors, 81 (31%) recruited patients for this study. Six hundred fifty seven neck pain patients provided baseline data with 274 being acute (< 4 weeks), 124 being subacute (4 – 12 weeks) and 255 being chronic (> 12 weeks). The 124 subacute patients in the database were not included in this study and 4 other patients were deleted from the database because they could not be reached for the three consecutive follow-up telephone calls. This resulted in baseline data for 529 total acute and chronic patients (Table 1). The mean age was 40.0 (SD ± 12.58) and 41.8 (±13.87) years for acute and chronic patients respectively. The majority of acute and especially chronic patients were female. Acute patients were significantly more likely to be in paid employment, to be taking pain medication and to be in ‘good health’ compared to chronic patients. There was a significant difference in the number of previous episodes of neck pain between the acute and chronic patients with chronic patients more likely to report four or more previous episodes (p = 0.001). There was no difference between acute and chronic patients for the proportion presenting with radiculopathy, pain onset due to trauma, smoking, or symptomatic for dizziness.

**Table 1:** Baseline characteristics for acute and chronic patients

	**Acute**	**Chronic**	**P-value**
	**(< 4 weeks)**	**(> 12 weeks)**	
	**(N = 274)**	**(N = 255)**	
Gender (Male)	112 (40.9)	89 (34.9)	0.157
Age (years)	40.0 (±12.58)	41.8 (±13.87)	0.114
In paid employment	235 (86.7)	200 (79.1)	0.020*
Taking pain meds	101 (37.0)	66 (25.9)	0.006*
Radiculopathy present	34 (12.5)	29 (11.4)	0.691
Previous episodes:			
None	109 (40.5)	116 (47.5)	0.0001*
1–3	82 (30.5)	25 (10.2)	
4 or more	78 (29.0)	103 (42.2)	
Trauma onset	32 (11.7)	40 (15.7)	0.179
Smoker	61 (22.8)	56 (22.6)	0.96
In good health	197 (73.0)	138 (54.4)	0.001*
Dizziness present	51 (44.7)	64 (45.1)	0.957

Values are numbers (%). N = number of observations. * = p < 0.05.

Table 2 compares the baseline NRS (neck and arm) scores as well as the BQN subscale and total scores for acute and chronic patients. Acute patients reported significantly higher baseline values for neck pain, disability in activities of daily living, disability in social activities and the total BQN score.

**Table 2:** Numerical rating scale (NRS) for pain and Bournemouth Questionnaire (BQ) baseline scores for acute and chronic patients

	**Acute baseline**	**Chronic baseline**	**p- Value**
	**(N =272)**	**(N = 255)**	
NRS neck	6.12 (±2.01)	5.5 (±2.29)	0.001*
NRS arm	2.28 (±2.20)	2.23 (±2.70)	0.838
BQ pain	5.91 (±2.34)	5.53 (±2.23)	0.06
BQ Disability in activities of daily living	4.81 (±2.86)	3.75 (±2.58)	0.0001*
BQ Disability in social activities	4.32 (±3.17)	3.03 (±2.95)	0.0001*
BQ Anxiety	5.41 (±2.88)	5.63 (±2.69)	0.366
BQ Depression	3.44 (±3.16)	3.41 (±3.04)	0.918
BQ Fear-avoidance beliefs	4.80 (±2.94)	4.43 (±2.93)	0.148
BQ Locus of control	4.80 (±2.94)	4.43 (±2.93)	0.091
BQ Total score	33.96 (±15.26)	30.50 (±14.18)	0.008*

Values are means (±SD). N = numbers of observations. * = p < 0.05.

### General outcomes

**Figure 1:**
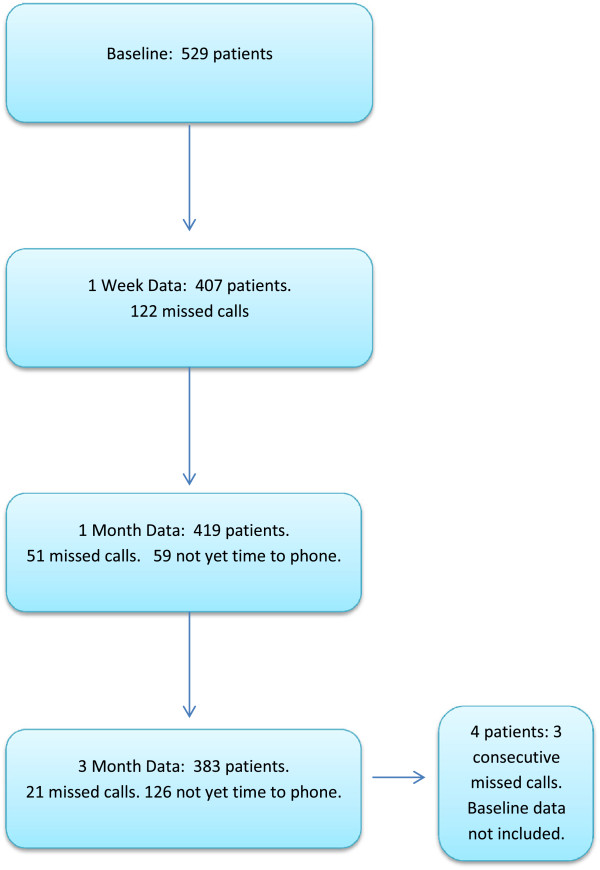
Flow chart of available patient data and source of missing data at each collection time point.

At 1 week after the start of treatment 77.8% of acute patients and 37.6% of chronic patients reported that they were significantly ‘improved’. By 1 month 86.6% of acute patients and 62.4% of chronic patients reported being ‘improved and at 3 months 84.3% of acute patients and 70.1% of chronic patients stated that they were ‘improved’. Actual ‘worsening’ of their condition was reported by less than 4% of acute patients and less than 9% of chronic patients at any time point. Figure 1 shows the number of patients with available data at each data collection time point as well as the sources of missing data. The reasons for the smaller sample sizes at the 1 week, 1 month and 3 month data collection time points are two fold. First, there is a narrow window within which these telephone calls are allowed in this study (e.g. between days 6 and 8 for 1 week data). If patients are not able to be contacted after several attempts during the data collection window, missing data are recorded for that time point but these patients remain in the study. However, if a patient cannot be contacted for 3 consecutive time points, they are removed from the study, including their baseline data. Second, this is still an ongoing study with new patients recruited weekly in order to establish a large database for future research. Thus for several of the patients with baseline and 1 week follow-up data, the dates for the 1 and 3 month follow-up telephone calls were not yet reached in the data captured in this particular study.

### Prognostic variables

#### Outcome at 1 Week

Univariate logistic regression analysis of potential predictors at baseline for improvement at 1 week for acute and chronic patients is shown in Table 3. No factors were associated with improvement for the acute patients and only 3 factors, namely the BQN pain and depression subscales as well as the total BQN score, were associated for improvement in the chronic patients. In view of a lack of strong association between baseline variables and early improvement at 1 week no further analysis was conducted.

**Table 3:** Univariate logistic regression analysis of potential predictors at baseline for improvement at 1 week in acute and chronic patients

**Predictor variable**	**Acute (N = 217)**		**Chronic (N = 190)**	
	**Unadjusted OR (95% CI)**	**p-value**	**Unadjusted OR (95% CI)**	**p-value**
Gender (male)	0.84 (0.44 to 1.61)	0.61	0.85 (0.46 to 1.59)	0.62
*Age (higher)	0.99 (0.97 to 1.02)	0.60	1.00 (0.98 to 1.02)	0.87
Dizziness present	1.63 (0.24 to 1.69)	0.36	1.08 (0.48 to 2.41)	0.86
Radiculopathy present	1.69 (0.55 to 5.18)	0.36	0.53 (0.18 to 1.52)	0.24
Trauma	0.91 (0.32 to 2.59)	0.86	0.90 (0.40 to 2.06)	0.81
Previous episodes category (higher)	1.10 (0.50 to 2.42)	0.82	1.23 (0.65 to 2.35)	0.52
Taking medication	0.86 (0.44 to 1.66)	0.64	1.38 (0.71 to 2.68)	0.35
Paid employment	1.01 (0.41 to 2.51)	0.98	1.00 (0.76 to 1.33)	0.981
Smoke	1.11 (0.50 to 2.43)	0.80	1.78 (0.87 to 3.61)	0.12
In good health	1.22 (0.61 to 2.46)	0.57	1.08 (0.60 to 1.96)	0.79
*BQ Pain	0.98 (0.85 to 1.12)	0.73	**1.13 (0.99 to 1.29)**	**0.08**
*BQ Disability	1.06 (0.94 to 1.18)	0.35	1.09 (0.97 to 1.23)	0.15
*BQ Social disability	1.08 (0.98 to 1.19)	0.14	1.07 (0.96 to 1.19)	0.21
*BQ Anxiety	1.02 (0.91 to 1.14)	0.74	1.10 (0.98 to 1.24)	0.11
*BQ Depression	0.99 (0.89 to 1.10)	0.83	**1.12 (1.01 to 1.24)**	**0.03**
*BQ Fear avoidance beliefs	0.96 (0.85 to 1.07)	0.44	1.09 (0.98 to 1.21)	0.69
*BQ Locus of control	1.05 (0.94 to 1.18)	0.37	0.98 (0.88 to 1.09)	0.69
*BQ Total	1.01 (0.98 to 1.03)	0.64	**1.02 (1.00 to 1.04)**	**0.07**
*NRS Neck	1.02 (0.98 to 1.03)	0.79	1.03 (0.91 to 1.17)	0.65
*NRS Arm	0.96 (0.86 to 1.08)	0.48	0.99 (0.88 to 1.12)	0.92

*Continuous variables. BQ = Bournemouth Questionnaire. NRS = Numerical rating scale. OR = odds ratio (95% Confidence intervals). N = Number of observations.

#### Outcome at 1 Month

Univariate logistic regression analysis was also carried out to determine the unadjusted associations between baseline variables and 1 week outcomes with improvement at 1 month for the acute and chronic patients (Table 4). For the acute patients, six variables were associated with improvement at 1 month. These were the ‘social disability’ subscale, ‘depression’ subscale, PGIC at 1 week, the baseline to 1 week ‘pain’ subscale change score, the baseline to 1 week ‘locus of control’ subscale change score, and the baseline to 1 week NRS neck pain change score.

**Table 4:** Univariate logistic regression analysis of potential predictors at baseline and 1 week for improvement at 1 month in acute and chronic patients

**Predictor variable**	**Acute (N = 215)**		**Chronic (N = 204)**	
	**Unadjusted OR (95% CI)**	**p-value**	**Unadjusted OR (95% CI)**	**p-value**
Gender (male)	0.69 (0.32-1.51)	0.36	0.75 (0.42 to 1.35)	0.34
*Age (higher)	0.98 (0.95 to 1.01)	0.13	1.00 (0.98 to 1.02)	0.85
Dizziness present	1.54 (0.47 to 5.02)	0.47	1.02 (0.45 to 2.31)	0.96
Radiculopathy present	2.19 (0.49 to 9.78)	0.30	0.60 (0.25 to 1.44)	0.25
Trauma	0.74 (0.24 to 2.36)	0.62	1.29 (0.59 to 2.83)	0.52
Previous episodes category (higher)	1.18 (0.47 to 2.93)	0.73	1.31 (0.71 to 2.43)	0.39
Taking medication	1.76 (0.74 to 4.18)	0.20	1.04 (0.54 to 2.00)	0.40
Paid employment	0.90 (0.29 to 2.78)	0.85	**0.55 (0.27 to 1.13)**	**0.10**
Smoke	1.43 (0.51 to 3.98)	0.49	1.01 (0.50 to 2.03)	0.98
In good health	1.72 (0.76 to 3.90)	0.19	1.58 (0.89 to 2.81)	0.12
*BQ Pain	1.09 (0.83 to 1.28)	0.31	1.00 (0.89 to 1.13)	0.98
*BQ Disability	1.08 (0.94 to 1.24)	0.29	1.01 (0.90 to 1.13)	0.87
*BQ Social disability	**1.12 (0.98 to 1.27)**	**0.09**	1.07 (0.97 to 1.19)	0.18
*BQ Anxiety	0.97 (0.85 to 1.12)	0.69	1.06 (0.75 to 1.17)	0.31
*BQ Depression	**0.90 (0.80 to 1.01)**	**0.08**	0.96 (0.88 to 1.06)	0.42
*BQ Fear avoidance beliefs	1.05 (0.92 to 1.20)	0.51	1.03 (0.94 to 1.14)	0.52
*BQ Locus of control	1.08 (0.95 to 1.24)	0.25	0.96 (0.87 to 1.07)	0.46
*BQ Total	1.01 (0.98 to 1.04)	0.49	1.00 (0.98 to 1.02)	0.83
*NRS Neck	1.08 (0.90 to 1.30)	0.39	1.00 (0.88 to 1.13)	0.93
*NRS Arm	1.05 (0.90 to 1.22)	0.53	0.98 (0.89 to 1.09)	0.76
**PGIC @ 1 week**	**4.04 (1.63 to 10.07)**	**.003**	**4.60 (2.04 to 10.38)**	**.0001**
*Baseline: 1 Week BQ Pain Change Score	**1.14 (0.99 to 1.32)**	**0.08**	1.01 (0.88 to 1.15)	0.88
*Baseline:1 Week BQ Disability Change Score	1.09 (0.95 to 1.26)	0.23	0.99 (0.88 to 1.12)	0.90
*Baseline: 1 Week BQ Social disability Change Score	1.10 (0.95 to 1.28)	0.20	1.10 (0.98 to 1.25)	0.12
*Baseline: 1 Week BQ Anxiety Change Score	1.13 (0.96 to 1.32)	0.14	1.08 (0.93 to 1.25)	0.31
*Baseline: 1 Week BQ Depression Change Score	0.99 (0.85 to 1.16 )	0.93	1.01 (0.90 to 1.14)	0.84
*Baseline: 1 Week BQ Fear avoidance beliefs Change Score	0.99 (0.86 to 1.16)	0.88	1.08 (0.96 to 1.22)	0.21
*Baseline: 1 Week BQ Locus of control Change Score	**1.12 (1.00 to 1.26)**	**0.05**	1.03 (0.94 to 1.13)	0.58
*Baseline: 1 Week BQ Total Change Score	1.02 (0.99 to 1.05)	0.13	1.01 (0.92 to 1.21)	0.43
*Baseline: 1 Week NRS Neck Change Score	**1.42 (1.14 to 1.77)**	**0.002**	1.06 (0.92 to 1.21)	0.43
*Baseline: 1 Week NRS Arm Change Score	1.12 (0.92 to 1.36)	0.27	0.95 (0.82 to 1.10)	0.49

*Continuous variables. BQ = Bournemouth Questionnaire. NRS = Numerical rating scale. OR = odds ratio (95% Confidence intervals). N = Number of observations. PGIC = Patient global impression of change scale.

Only two variables in the univariate logistic regression analysis were associated with 1 month improvement for the chronic patients. These were being in paid employment and the PGIC score at 1 week.

The subsequent multivariate analysis for predictors of improvement at 1 month resulted in four variables independently associated with the outcome for the acute patients (Table 5). These were the PGIC at 1 week, baseline to 1 week NRS neck pain change score, baseline to 1 week BQ ‘pain’ subscale change score and the baseline ‘depression’ subscale score. Acute patients who reported significant improvement at 1 week on the PGIC were almost 3 times more likely to report improvement at 1 month. For every 1 point change (decrease) on the baseline to 1 week BQ ‘pain’ subscale score, acute patients were approximately 20% more likely to be improved at 1 month. For every 1 point change (decrease) in the baseline to 1 week NRS neck pain score, these patients were 36% more likely to be improved at 1 month. For every 1 point increase in the baseline ‘depression’ subscale score, acute patients were 16% less likely to report improvement at 1 month. This final model explained 21.7% of the variance in improvement at 1 month.

**Table 5:** Multivariate logistic regression analysis of prognostic predictors at baseline and at 1 week for improvement at 1 month in acute and chronic patients

	**Coefficient**	**OR**	**95% CI**	**p-value**	**Sensitivity; Specificity; Percentage correctly predicted; Area under ROC (95% CI); Adjusted R**^**2**^
**Acute N = 180**					
BQ 5: Baseline Depression	−0.18	0.84	0.71 to 0.98	0.024	99.4%; 4.3%; 87.2%; 0.79 (0.70 to 0.88); 21.7%
Baseline to 1 Week BQ Pain change score	0.19	1.21	1.02 to 1.44	0.032	
Baseline to1 Week NRS Neck Change Score	0.31	1.36	1.10 to 1.75	0.017	
PGIC @ 1 Week	1.05	2.87	1.08 to 7.64	0.035	
**Chronic N = 156**					
PGIC @ 1 Week	1.48	4.41	1.95 to 9.96	0.001	100.0%; 0%; 67.3%; 0.66 (0.57 to 0.75); 12.7%

BQ = Bournemouth Questionnaire. NRS = Numerical pain rating scale. OR = odds ratio (95% Confidence intervals). N = Number of observations. PGIC = Patient global impression of change scale.

Only 1 factor, the PGIC at 1 week, remained independently predictive of improvement in chronic patients at 1 month. Patients improved at 1 week were approximately 4 times more likely to be improved at 1 month. However, the final model explained very little (12.7%) of the variance in improvement at 1 month.

### Outcome at 3 months

Table 6 shows the univariate logistic regression analyses to determine the associations between baseline variables, 1 week outcomes and 1 month outcomes and improvement at 3 months for both acute and chronic patients. Sixteen variables were predictors of improvement for acute patients and eight variables for chronic patients.

**Table 6:** Univariate logistic regression analysis of potential predictors at baseline, at 1 week and at 1 month for improvement at 3 months in acute and chronic patients

**Predictor variable**	**Acute (N = 197)**		**Chronic (N = 185)**	
	**Unadjusted OR (95% CI)**	**p-value**	**Unadjusted OR (95% CI)**	**p-value**
Gender (male)	1.31 (0.60-2.85)	0.50	0.63 (0.33 to 1.23)	0.18
*Age (higher)	0.98 (0.96 to 1.01)	0.29	1.00 (0.98 to 1.02)	0.99
Dizziness present	1.40 (0.42 to 4.65)	0.58	1.06 (0.42 to 2.67)	0.90
Radiculopathy present	0.89 (0.31 to 2.54)	0.82	1.34 (0.46 to 3.89)	0.59
Trauma	0.87 (0.36 to 2.12)	0.76	0.78 (0.34 to 1.82)	0.56
Previous episodes category (higher)	0.65 (0.30 to 1.39)	0.27	1.41 (0.71 to 2.78)	0.33
Taking medication	0.65 (0.30 to 1.39)	0.27	0.89 (0.4 to 1.82)	0.75
Paid employment	1.30 (0.45 to 3.75)	0.63	0.61 (0.28 to 1.34)	0.22
Smoke	1.88 (0.62 to 5.72)	0.27	0.96 (0.45 to 2.08)	0.93
In good health	1.42 (0.63 to 3.18)	0.40	0.74 (0.39 to 1.42)	0.37
*BQ Pain	0.98 (0.82 to 1.15)	0.77	1.00 (0.87 to 1.15)	0.98
*BQ Disability	1.10 (0.96 to 1.26)	0.18	1.05 (0.92 to 1.219)	0.49
*BQ Social disability	**1.13 (0.99 to 1.28)**	**0.06**	1.09 (0.97 to 1.22)	0.16
*BQ Anxiety	1.02 (0.89 to 1.17)	0.76	**1.12 (0.99 to 1.26)**	**0.08**
*BQ Depression	0.91 (0.81 to 1.02)	0.11	0.97 (0.88 to 1.08)	0.63
*BQ Fear avoidance beliefs	**1.14 (0.99 to 1.30)**	**0.07**	1.02 (0.91 to 1.14)	0.70
*BQ Locus of control	1.02 (0.89 to 1.17)	0.77	1.03 (0.91 to 1.15)	0.66
*BQ Total	1.01 (0.98 to 1.04)	0.47	1.01 (0.99 to 1.04)	0.37
*NRS Neck	1.09 (0.91 to 1.30)	0.36	1.02 (0.89 to 1.18)	0.73
*NRS Arm	**0.90 (0.79 to 1.02)**	**0.09**	1.901 (0.89 to 1.14)	0.93
PGIC @ 1 week	**3.61 (1.47 to 8.89)**	**0.005**	**3.37 (1.42 to 8.00)**	**0.006**
*Baseline: 1 Week BQ Pain Change Score	1.13 (0.96 to 1.32)	0.15	1.06 (0.91 to 1.24)	0.43
*Baseline: 1 Week BQ Disability Change Score	1.05 (0.91 to 1.21)	0.49	0.98 (0.85 to 1.13)	0.79
*Baseline:1 Week BQ Social Disability Change Score	1.06 (0.92 to 1.22)	0.42	0.99 (0.87 to 1.12)	0.83
*Baseline: 1 Week BQ Anxiety Change Score	1.06 (0.92 to 1.23)	0.42	1.12 (0.98 to 1.27)	0.11
*Baseline: 1 Week BQ Depression Change Score	0.89 (0.77 to 1.04)	0.14	1.09 (0.95 to 1.24)	0.21
*Baseline: 1 Week BQ Fear avoidance beliefs Change Score	**1.20 (1.02 to 1.40)**	**0.026**	0.98 (0.87 to 1.12)	0.81
*Baseline: 1 Week BQ Locus of control Change Score	1.06 (0.95 to 1.19)	0.29	1.00 (0.90 to 1.12)	0.96
*Baseline: 1 Week BQ Total Change Score	1.02 (0.99 to 1.05)	0.24	1.01 (0.98 to 1.05)	0.38
*Baseline: 1 Week NRS Neck Change Score	**1.25 (1.04 to 1.51)**	**0.02**	1.10 (0.87 to 1.17)	0.94
*Baseline: 1 Week NRS Arm Change Score	0.94 (0.79 to 1.12)	0.48	1.03 (0.87 to 1.22)	0.72
PGIC @ 1 Month	**3.94 (1.47 to 10.60)**	**0.007**	**5.82 (2.83 to 11.97)**	**0.001**
*Baseline: 1 Month BQ Pain Change Score	**1.30 (1.11 to 1.52)**	**0.001**	**1.22 (1.06 to 1.41)**	**0.006**
*Baseline: 1 Month BQ Disability Change Score	**1.41 (1.18 to 1.68)**	**0.001**	**1.15 (1.02 to 1.30)**	**0.02**
*Baseline:1 Month BQ Social Disability Change Score	**1.44 (1.22 to 1.71)**	**0.001**	1.06 (0.95 to 1.18)	0.33
*Baseline: 1 Month BQ Anxiety Change Score	**1.26 (1.09 to 1.46)**	**0.001**	**1.16 (1.04 to 1.30)**	**0.008**
*Baseline: 1 Month BQ Depression Change Score	**1.15 (0.99 to 1.33)**	**0.08**	1.05 (0.94 to 1.19)	0.40
*Baseline: 1 Month BQ Fear avoidance beliefs Change Score	**1.33 (1.14 to 1.55)**	**0.001**	1.04 (0.94 to 1.14)	0.49
*Baseline: 1 Month BQ Locus of control Change Score	**1.36 (1.16 to 1.60)**	**0.001**	**1.18 (1.07 to 1.30)**	**0.001**
*Baseline: 1 Month BQ Total Change Score	**1.08 (1.04 to 1.11)**	**0.001**	**1.03 (1.01 to 1.06)**	**0.004**
*Baseline: 1 Month NRS Neck Change Score	**1.30 (1.10 to 1.54)**	**0.002**	**1.18 (1.03 to 1.36)**	**0.02**
*Baseline: 1 Month NRS Arm Change Score	1.10 (0.93 to 1.31)	0.26	1.12 (0.94 to 1.33)	0.22

*Continuous variables. BQ = Bournemouth Questionnaire. NRS = Numerical rating scale. OR = odds ratio (95% Confidence intervals). N = Number of observations. PGIC = Patient global impression of change.

In the subsequent multivariate model for improvement at 3 months, only two variables remained as independent predictors in the acute patients and one variable in the chronic patients (Table 7). The PGIC score at 1 week and the baseline to 1 month BQN total change score were independent predictors in acute patients and the PGIC at 1 month was the only predictor of improvement at 3 months in the chronic patients. Acute patients who reported being improved on the PGIC at 1 week were approximately 3 times more likely to be improved at 3 months. For every 1 point change (decrease) in the BQN baseline to 1 month total score, acute patients were 7% more likely to be improved at 3 months. Chronic patients who reported being improved on the PGIC at 1 month were 6 times more likely to be improved at 3 months. The models explained 28.8% and 19.9%% of the variance in outcome at 3 months for acute and chronic patients respectively.

**Table 7:** Multivariate logistic regression analysis of prognostic predictors at baseline, at 1 week and at 1 month for improvement at 3 months

	**Coefficient**	**OR**	**95% CI**	**p-value**	**Sensitivity; Specificity; Percentage correctly predicted; Area under ROC (95% CI); Adjusted R**^**2**^
Acute N = 146					
Baseline to 1 Month BQN total Change Score	0.66	1.07	1.03 to 1.11	0.001	97.6%; 35.0%; 89%; 0.82 (0.72 to 0.92); 28.8%
PGIC @ 1 Week	1.21	3.35	1.13 to 9.92	0.029	
ChronicN = 133					
PGIC @ 1 Month	1.80	6.04	2.76 to 13.69	0.001	77.9%; 63.2%; 73.7%; 0.71 (0.62 to 0.79); 19.9%

BQ = Bournemouth Questionnaire. PGIC = Patient global impression of change. N = number of observations.

## Discussion

The predictors of improvement in acute and chronic neck pain patients undergoing chiropractic treatment appear to be very similar to those found for patients suffering from low back pain [18-25]. The most consistent predictor of improvement for both acute and chronic neck pain patients was reported prior improvement. Acute patients who reported being improved 1 week after the start of treatment were approximately 3 times more likely to be improved at 3 months while chronic neck pain patients who reported being improved at 1 month were over 6 times more likely to also report improvement at 3 months.

Although the various demographic details collected at baseline in this study were largely not predictive of improvement at 1 week, 1 month or 3 months, it is important to emphasize two clinical conditions that were not predictive of a worse outcome. Thus neck pain patients who also had cervical radiculopathy (34 acute and 29 chronic patients) or who reported associated dizziness (51 acute and 64 chronic patients) improved as much as neck pain patients without these additional signs or symptoms. Previous studies on low back pain patients undergoing chiropractic treatment reported that the presence of additional leg pain was a negative predictor of improvement [25-27]. The Swiss chiropractors contributing patients to this current study were specifically informed prior to the start of data collection that the presence of arm pain alone was not sufficient to diagnose radiculopathy and that clinical signs of nerve root compression were also required. However, it was left up to the individual chiropractor to make this diagnosis based on clinical experience. There was no attempt to determine whether or not their diagnosis was correct. Swiss chiropractors have a significant amount of exposure to patients with radiculopathy in their mandatory two year post-graduate program and were therefore assumed competent in their diagnosis.

Acute neck pain sufferers (symptoms < 4 weeks) presented with higher levels of pain and disability compared to chronic patients (symptoms > 12 weeks), but improved more quickly and in a higher proportion than chronic patients. Although the acute patients did better than the chronic patients (in part due to the natural history of neck pain), it is important to point out that many of the chronic patients also improved. Additionally, the percentage of chronic patients reporting improvement continued to rise at each data collection time point whereas this appeared to level off at 1 month for acute patients. This study intentionally analyzed acute and chronic patients separately rather than including duration of neck pain into the prediction model. Previous research has already shown that duration of neck pain is a strong predictor of outcome [7] and it was particularly desired to assess the outcomes of chronic patients as they are the ones who are often more challenging and expensive to treat.

Although there was no significant age difference between the acute and chronic neck pain patients in this study, the chronic patients were significantly less likely to be in paid employment, reported significantly more prior episodes of neck pain and were less likely to be in good general health. The duration of symptoms and the number of previous episodes of neck pain have been found to be predictors of outcome in previous studies [7,8,14]. Surprisingly, this study did not find that the number of previous episodes of neck pain was linked with improvement as reported by Rubinstein et al. [7]. However, there was a difference between the Rubinstein et al. study and this current study in the way previous neck pain episodes were classified. This variable was dichotomized (yes/no) in the Rubenstien et al. study compared to categorizing previous episodes into one of three groups (none, 1–3, 4 or more) in this current study. It was hoped that providing more specific detail about the number of previous episodes would prove to be predictive of improvement. However, this was not the case. Also as reported in other studies, the majority of patients in this study were women, particularly in the chronic category [1,3]. This may be one reason why chronic patients were less likely to be in paid employment. Women, particularly with children, are less likely to work outside of the home in Switzerland compared to some other western countries [28].

### Limitations

There are several limitations to this study. Although a high proportion of both acute and chronic neck pain patients improved at all time points, without a control group this improvement cannot be attributed to treatment. It is also acknowledged that the improvement for many of the acute patients was most likely due to natural history. In this study we collected baseline variables using paper questionnaires but subsequent outcomes were collected through telephone interviews. It has been shown that telephone interview data may have a slightly positive effect on outcomes encouraging patients to report more favorably [29-31]. An attempt was made to minimize this effect by employing anonymous research assistants, unknown to the patients, who collected the telephone data from the university rather than from the practice setting where the patient was being treated.

Data was also purposely not collected on specific treatments applied or frequency of chiropractic treatment. It would be interesting and important for the education of chiropractors to compare treatment and technique methods with patient outcomes in the future. Data was collected in this study on the date of the last treatment however, and this will be evaluated and reported when the 1 year outcomes data is assessed in the near future.

Another limitation to the study may be the validity of the Bournemouth Questionnaire to adequately measure the psychosocial domains such as depression, anxiety, etc. as these variables were not predictive of improvement at 3 months. However, when compared to the NPAD and NDI the BQ was much more sensitive to change on all domains [16], including the domains of anxiety, depression, fear avoidance and pain locus of control. Certainly ‘depression’ was identified as a negative predictor of improvement at 1 month in the multivariate analysis for acute patients which suggests that for depression, at least, the BQN is sensitive as a predictor of outcome.

A further limitation may be the fact that most of the variables that were not continuous variables were dichotomized into yes/no or present/absent with the exception of ‘number of previous episodes’ and ‘general health’. This was done to facilitate the clinical utility of this study and assist in interpretation of the results. In most cases there was a straightforward yes/no (i.e. smoking, paid employment, etc.) answer. In those few variables where this was not the case, we used clinical experience to make the decision and it is fair to say that we may have made the wrong judgment on those two variables which were categorized with three options. This is the same model used in a similar research study done on low back pain patients however [18].

Although this study found that neither the presence of radiculopathy nor dizziness were negative predictors of outcome, no power analysis was done to determine whether the 115 patients with dizziness or the 63 patients with radiculopathy were adequate sample sizes to definitively draw these conclusions. However, at least for the dizziness patients, 115 is approximately one fifth of the total sample of 529 patients.

Previous research has identified ‘stiffness’ as a predictor of outcome (8) in neck pain patients treated with SMT. This was not included in the baseline evaluations done by the treating chiropractors and is therefore another limitation in this study.

The final possible limitation to this study may be the fact that compared to baseline there were fewer patients at the 1 and 3 month analyses. This was primarily due to the fact that it was not yet time for the 1 month and 3 month telephone calls for several patients. However, both the acute and chronic groups at 3 months had nearly 200 patients each. The first author of this study is directly in charge of large medical databases in two departments at this hospital. Experience of periodically calculating and monitoring pain scores, PGIC scores, proportions of patients improving or worsening with various treatments in these databases has consistently demonstrated that once a threshold of approximately 100 patients in a cohort is reached, additional patients make minimal difference in the statistical results calculated. Therefore this missing data is unlikely to have changed the outcomes with sample sizes this large. If outcome data was available for any of the follow-up time periods, the patient and their baseline data remained in this study.

## Conclusions

For the most part, baseline variables were not predictive of outcome in neck pain patients and as a consequence prediction of outcome at 1 week was not possible using the potential predictors measured in this study. Instead, the most consistent predictor of improvement at both 1 and 3 months for both acute and chronic neck pain patients undergoing chiropractic treatment is significant self-reported improvement early on. Importantly, the co-existence of cervical radiculopathy or dizziness in addition to neck pain was not associated with a negative outcome for either acute or chronic patients.

## Competing interests

The authors declare that they have no competing interests.

## Authors’ contributions

CP: Concept and design of the study, collection and entry of data, analysis and interpretation of data, drafting and revising the manuscript, final approval of the manuscript. JB: Concept and design of the study, data analysis and interpretation, revising the manuscript, final approval of the manuscript. BKH: Concept and design of the study, data analysis, revising the manuscript, final approval of the manuscript. All authors read and approved the final manuscript.
